# A new approach for the determination of sunscreen levels in seawater by ultraviolet absorption spectrophotometry

**DOI:** 10.1371/journal.pone.0243591

**Published:** 2020-12-16

**Authors:** Antonio Tovar-Sánchez, Erica Sparaventi, Amandine Gaudron, Araceli Rodríguez-Romero

**Affiliations:** 1 Department of Ecology and Coastal Management, Institute of Marine Sciences of Andalusia, ICMAN (CSIC), Cádiz, Spain; 2 Department of Analytical Chemistry, Faculty of Marine and Environmental Sciences, University of Cádiz, Cádiz, Spain; VIT University, INDIA

## Abstract

Sunscreen is released into the marine environment and is considered toxic for marine life. The current analytical methods for the quantification of sunscreen are mostly specific to individual chemical ingredients and based on complex analytical and instrumental techniques. A simple, selective, rapid, reproducible and low-cost spectrophotometric procedure for the quantification of commercial sunscreen in seawater is described here. The method is based on the inherent properties of these cosmetics to absorb in the wavelength of 300–400 nm. The absorption at 303 nm wavelength correlates with the concentration of most commercial sunscreens. This method allows the determination of sunscreens in the range of 2.5–1500 mg L^-1^, it requires no sample pretreatment and offers a precision of up to 0.2%. The spectrophotometric method was applied to quantify sunscreen concentrations at an Atlantic Beach with values ranging from 10 to 96.7 mg L^-1^ in the unfiltered fraction and from the undetectable value to 75.7 mg L^-1^ in the dissolved fraction. This method is suggested as a tool for sunscreen quantifications in environmental investigations and monitoring programs.

## 1. Introduction

Among the many chemicals and emerging pollutants used by modern society, the marine environmental impacts of sunscreen products have attracted scientific and social attention in the past few years [1]. Every year, cosmetic companies flood the market with new sunscreens with different formulations and rheology (e.g. creams, oils, sprays, etc.). Sunscreens are a cocktail of chemicals that, when applied on our skin, protect against the harmful effects of the radiation of the sun. Therefore, in addition to emollients, emulsifiers, perfumes, and many other chemicals, they include UV filters (organic and inorganic) as active components that absorb, reflect or scatter UV radiation in the range of 400–320 nm (UVA) and/or 320–280 nm (UVB) [2]. Currently, there are around 50 organic UV-filters (e.g. derivatives of benzophenone, camphor, p-aminobenzoic acid, etc.), and 2 inorganic UV-filters (TiO_2_ and ZnO), allowed in sunscreen formulations [2, 3] by different legislations.

Sunscreen products reach the marine environment mainly by direct release through people swimming in the sea and through Waste Water Treatment Plants (WWTP) effluents, since many daily activities, such as showering, laundering or urinating are sources of UV-filters discharged to the WWTP, where they are not completely removed [4, 5]. UV-filters have been detected in many coastal matrices (i.e. seawater, beach sand, aquatic biota, etc.) [5–9] and have been demonstrated to cause a variety of different biological and toxicological responses in marine organisms affecting survival, behavior, growth, development and reproduction [10–16]. As a direct consequence of the detrimental impacts of some organic UV filters on many marine organisms (such as algae, coral, mussels, sea urchins, fish, dolphin) and fragile marine ecosystems (such as coral reefs) [17], some regions (i.e. Hawaii, and Key West in the USA; Bonaire island and the Pacific nation of Palau) have passed a bill banning sunscreens containing specific organic UV filters (e.g. oxybenzone and octinoxate). However, the environmental impact of sunscreens is not only due to the active ingredients (i.e. UV-filters) since many other compounds, such as inorganic nutrients and trace metals, are also released when the cosmetic comes into contact with seawater [18]. Therefore, the evaluation of their impact on the marine environment remains little studied because of the complex matrix of sunscreens and their multiple and unknown commercial formulations and the investigations are based on lab experiments and focused on a single ingredient, mainly UV filters [19, 20]. Consequently, the analytical methodologies involved in these studies are largely based on the quantification of their individual ingredients. Even in those studies where the effect of commercial sunscreens themselves are tested, the analytical methods used for their quantification are focused on the determination of some of their UV filters [2]. These current approaches do not lead us to estimate the exact quantity of these cosmetic cocktails in marine waters accurately and therefore predict their potential associated effects in the ecosystem. To date, the quantification of UV filters (organic and inorganic) in environmental samples involves sophisticated instrumentation (e.g. HPLC, GC, ICPMS) and sample pretreatment (e.g. preconcentration, salt removal, acid digestion, etc.) [2]. A non-destructive and relatively cheap technique as ATR-FTIR has been demonstrated to be valid for the quantification of sunscreen products, however its applicability has been focused in forensic investigations [21]. Here, we propose a simple spectrophotometric procedure for the quantification of commercial sunscreens in seawater. The method is based on the properties of these cosmetics to absorb UV radiation; it requires no sample pretreatments and the results are very simple and rapid for laboratory and on-board analysis. This method offers a valuable tool to understand the impact of sunscreens on the marine coastal ecosystems better, where information about concentration, distribution, transport and aging of sunscreen in seawater is lacking but crucial to evaluate the real impact of these emerging pollutants on our coasts.

## 2. Materials and methods

### 2.1 Apparatus

A Thermo Scientific^™^ GENESYS^™^ 60S UV-Vis Spectrophotometer equipped with a quartz cell with a 10 mm pathlength (PerkinElmer^™^) was used in this study. The VISIONlite^™^ Spectrophotometer Software (Thermo Scientific^™^) was used for scanning and data processing.

### 2.2 Commercial sunscreens and standard preparation

A total of 22 commercial sunscreens with different UV filters (i.e. organic and/or inorganic), texture (e.g. Gel Cream, Sun Milk, Spray, etc.) and solar protection factors (SPF) were used in this study (Table 1). Seawater (filtered through a 0.45 μm pore size filter) used for the dilutions were obtained from coastal well marine water (pH: 7.9–8.1 and salinity: 36–38). To determine the absorption spectra, 5 mg of each sunscreen were diluted in 50 mL of seawater.

**Table 1:** Properties of selected sunscreens: Texture indicated by the brand; Sun Protection Factor (SPF); chemical UV filters used; wavelength of maximum absorbance (λmax), and percentage of absorbance measured at 303 nm regarding maximum absorbance.

Sunscreen	Texture	SPF	UV filters*	λmax (nm)	Abs-303nm / Abs-λmax (%)	1	Octocrylene
1	Spray	10	1, 2, 3, 4, 15	363	94.9	2	Methylene bis-benzotriazolyl tetramethylbutylphenol
2	Spray Milk	15	1, 3, 5, 6	301	99.9	3	Bis-ethylhexyloxyphenol methoxyphenyl triazine
3	Gel Cream	15	1, 4, 7	316; 318	97.2	4	Butyl methoxydibenzoylmethane
4	Sun Milk	30	1, 4, 8, 15	303	100	5	Ethylhexyl methoxycinnamate
5	Spray	30	1, 4, 8	303	100	6	Methylene bis-benzotriazolyl tetramethylbutylphenol
6	Spray	30	1, 3, 4, 8, 9, 10, 15	344	79.3	7	Diethylhexyl butamido triazone
7	Cream	30	1, 7, 16	303	100	8	Ethylhexyl salicylate
8	Sun Milk	30	1, 3, 4, 15	303	100	9	Ethylhexyl triazone
9	Sun Milk	50+	1, 3, 4, 15	303	100	10	Drometrizole trisiloxane
10	Spray	50+	1, 3, 4, 8, 9, 10, 15	345	60.3	11	Avobenzone
11	Cream	50+	1, 3, 4, 8, 15	302	99.8	12	Homosalate
12	Sun Milk	50+	1, 3, 4, 15	300	97.9	13	Octisalate
13	Sun Milk	50+	1, 3, 4, 15	303	100	14	Trisiloxane
14	Foam	50	1, 4, 10	369	93.0	15	Titanium dioxide
15	Spray	30	1, 4, 9	347	90.7	16	Zinc oxide
16	Fluid	50+	2, 3, 7	315	95.6		
17	Gel	90	1, 9, 4, 16	320	96.2		
18	Sun Milk	50+	1, 3, 5, 9, 10, 15	344; 345	66.2		
19	Milk	50+	1, 4, 15	320; 321	92.9		
20	Cream	50	11, 12, 13, 14	323	95.8		
21	Gel	50	1, 5, 15, 16	315	97.1		
22	Gel Cream	25	1, 4, 7	316; 318	98.0		
Sunscreen Standard Stock	--	--	All	303	100		

* UV filters: 1 (Octocrylene); 2 (Methylene bis-benzotriazolyl tetramethylbutylphenol); 3 (Bis-ethylhexyloxyphenol methoxyphenyl triazine); 4 (Butyl methoxydibenzoylmethane); 5 (Ethylhexyl methoxycinnamate); 6 (Methylene bis-benzotriazolyl tetramethylbutylphenol); 7 (Diethylhexyl butamido triazone); 8 (Ethylhexyl salicylate); 9 (Ethylhexyl triazone); 10 (Drometrizole trisiloxane); 11 (Avobenzone); 12 (Homosalate); 13 (Octisalate); 14 (Trisiloxane); 15 (Titanium dioxide); 16 (Zinc oxide).

The calibration curves were prepared from a sunscreen standard stock containing a mix of 0.5 g of all selected sunscreens. Then, different aliquots of this stock were appropriately diluted with seawater to prepare the standards of the calibration curve.

### 2.3 Field and swimming pool sampling

**Fig 1:**
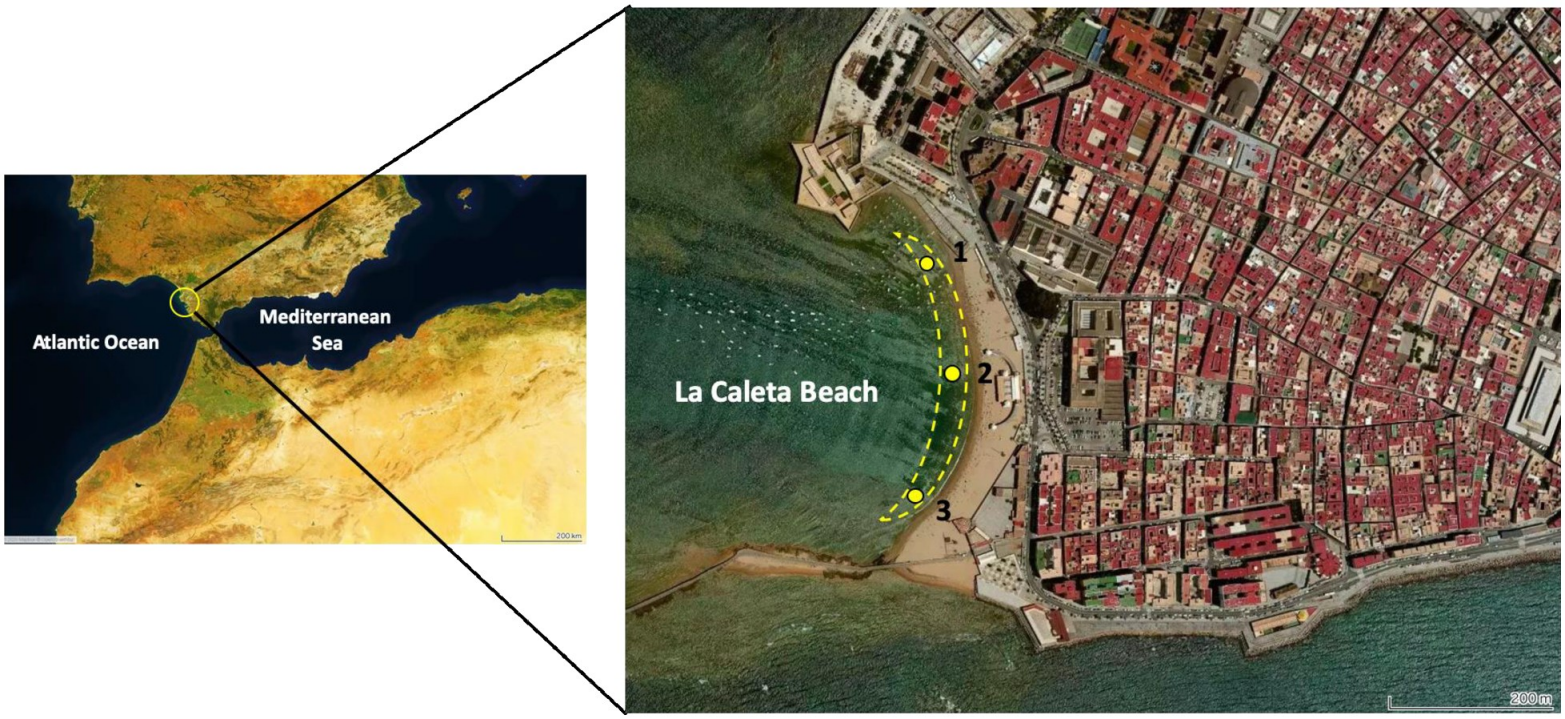
Sampling locations (yellow dots) at La Caleta Beach (June 28^th^, 2020). Yellow dashes indicate the bathing area on the sampling day (3000 m^2^). Images have been created using Tableau Public Softaware.

Coastal seawater samples were collected from La Caleta Beach (South of Spain, Cádiz, Fig 1) on May 28^th^, 2020 (during the confinement of the population due to the COVID-19 pandemic, and therefore without beachgoers) and on June 28^th^, 2020 (with the beach open to the population and without any restrictions). Sampling in a swimming pool (60 m^3^ of chlorinate water) was also collected on June 28^th^, 2020. Unfiltered (total fraction) and filtered (dissolved fraction) surface (5 cm depth) samples were collected in 13 mL polystyrene tubes at 3 nearshore locations along the beach and at one point in the swimming pool. Unfiltered samples were collected directly using a plastic syringe and filtered samples were collected using 0.45 μm nylon syringe filters. Samples were transported in the dark to the laboratory and measured within 12 hours after collection.

Absorbances of aliquots of standards and samples were directly measured in the 1 cm quartz cell against blanks (i.e. seawater from well for beach samples and ultrapure distilled water for swimming pool samples).

## 3. Result and discussion

### 3.1 Identification of the representative peak of absorption

**Fig 2:**
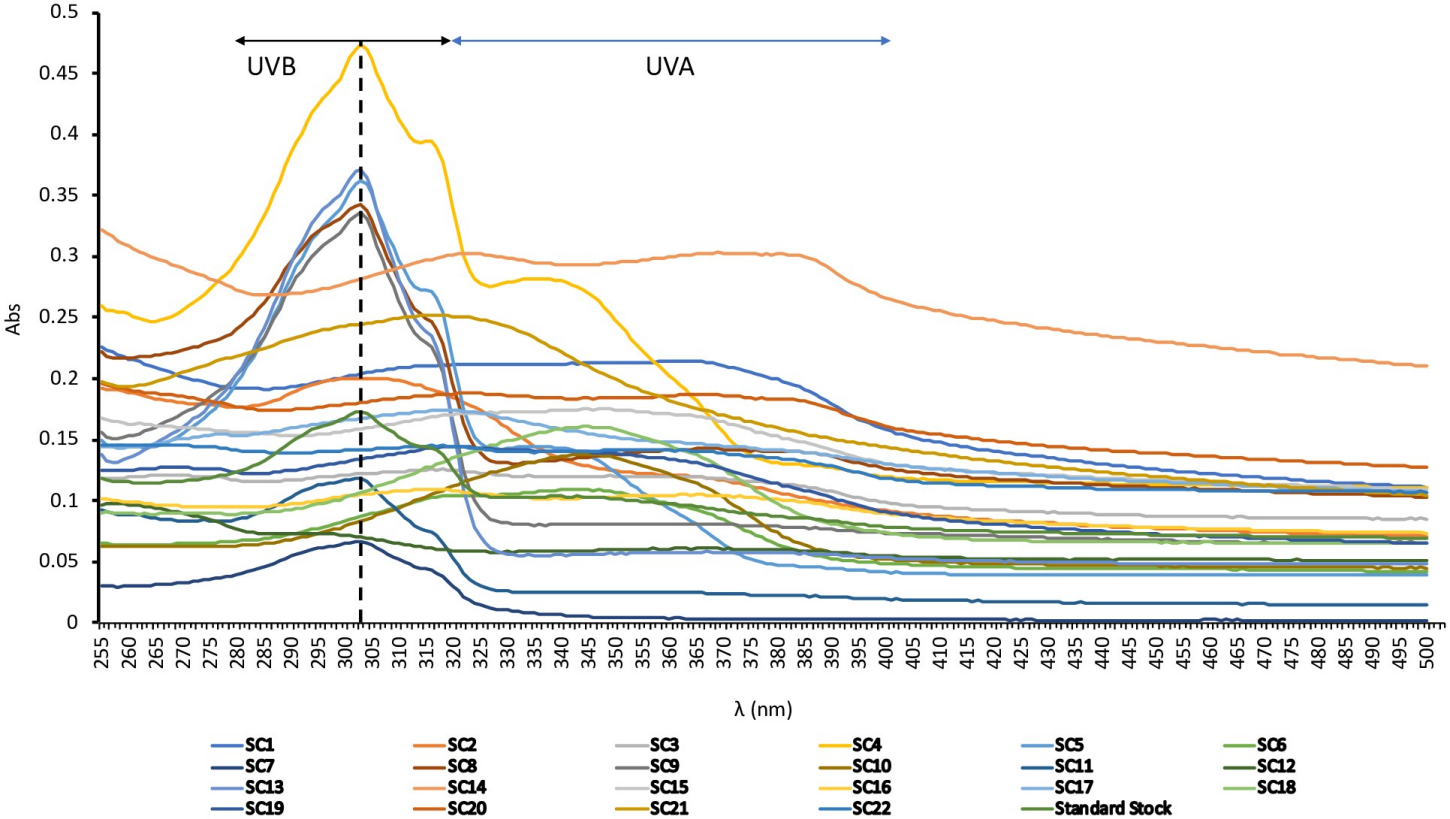
Absorption spectra of sunscreens. Grey and blue shaded areas include the UVB and UVA ranges of wavelength, respectively. Dashed line indicates the 303 nm wavelength.

Sunscreens are specifically manufactured to protect against UV radiation. Each company uses its own formulation to do this using different kinds and quantities of organic and/or inorganic UV filters are added in order to block a specific range of UV radiation. Fig 2 shows the spectra of absorbances in the range of 255–500 nm of 100mg L^-1^ of the 22 commercial sunscreens tested.

70% of sunscreens showed the maximum absorbance in the range of UVB (280–320 nm) and the remaining 30% sunscreens showed the maximum absorbance in the UVA range (320–400 nm) (Fig 2 and Table 1). Statistically (i.e. using the mode), the most frequent wavelength of maximum absorbance (λmax) measured was 303 nm, with up to 6 sunscreens having their maximum absorbances at that wavelength (Table 1). The absorbances measured at 303 nm for the rest of sunscreens, represented between 60% (for sunscreen 10) and 99% (for sunscreen 11) of their maximum absorbances. The highest peak of absorbance of the sunscreen standard stock solution (composed of a mixture of all sunscreens) was also measured at 303 nm confirming that this wavelength seems to be the one that best represents the levels of sunscreen in the sea (Fig 2 and Table 1).

### 3.2 Analytical figures validating the proposed spectrophotometric procedure

**Fig 3:**
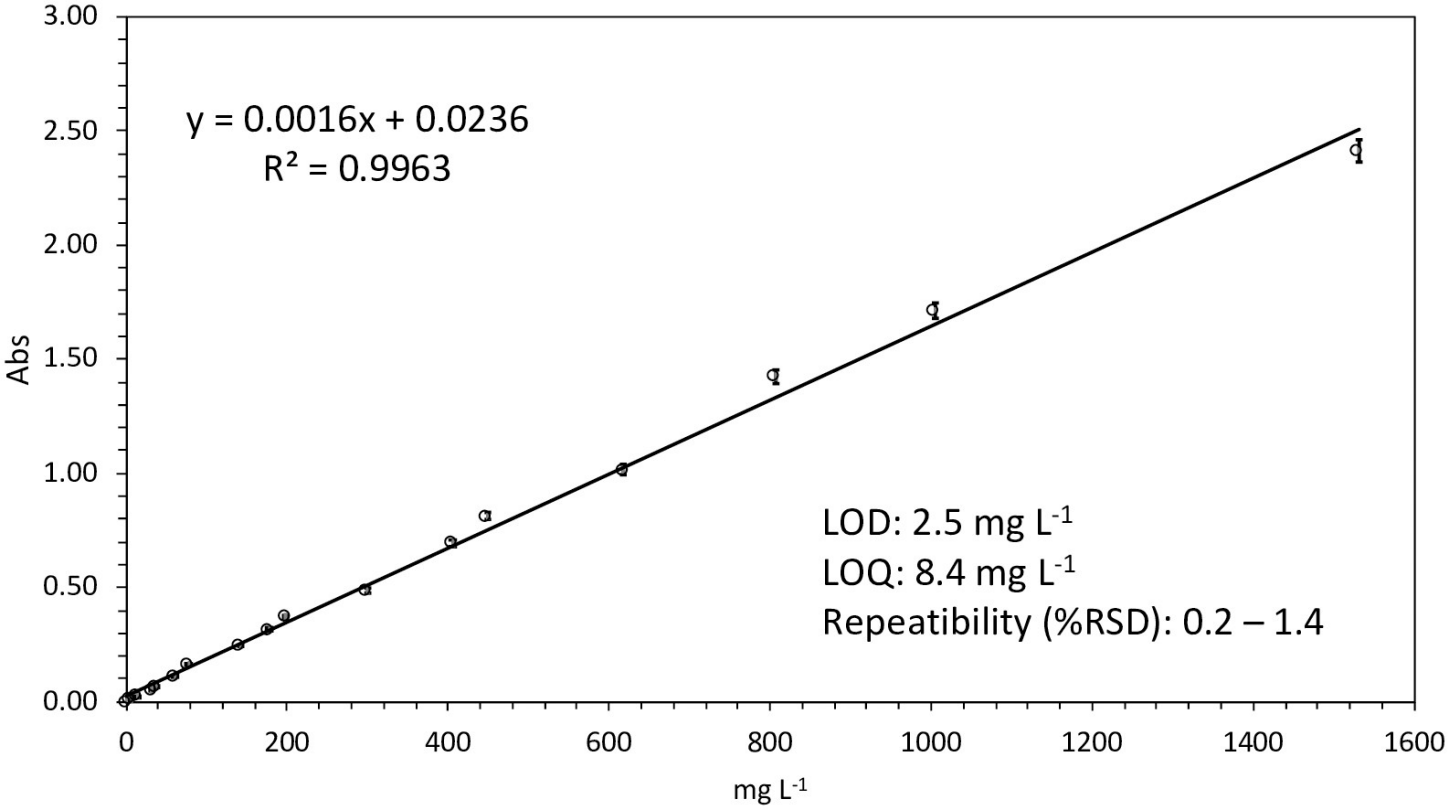
Sunscreen calibration curve and equation. Working range: 0–1500 mg L^-1^, number of standards 17 (measured in triplicate).

A calibration curve using the standard sunscreen stock was prepared for the determination, at the 303 nm wavelength, of the concentrations of sunscreens in seawater. The method shows a high linearity with regression coefficients (R^2^) > 0.996 and employed a working range set from 6 to 1500 mg L^-1^ (Fig 3). The limits of detection (LOD), calculated as 3(S_y_/S) criteria, where S_y_ is the standard deviation of the response of the calibration curve and S (the slope of the calibration curve) was 2.5 mg L^-1^. The limits of quantification (LOQ), calculated as 10(S_y_/S) criteria, was 8.4 mg L^-1^. The repeatability, expressed as relative standard deviation (RSD), was evaluated by measuring the absorbance at the 303 nm wavelength selected of three standard replicates of seawater solutions at three different concentrations (5, 100 and 500 mg L^-1^). The values were between 0.2 and 1.4%, showing the accuracy of the method.

### 3.3 Conservation and aging of the standard

**Fig 4:**
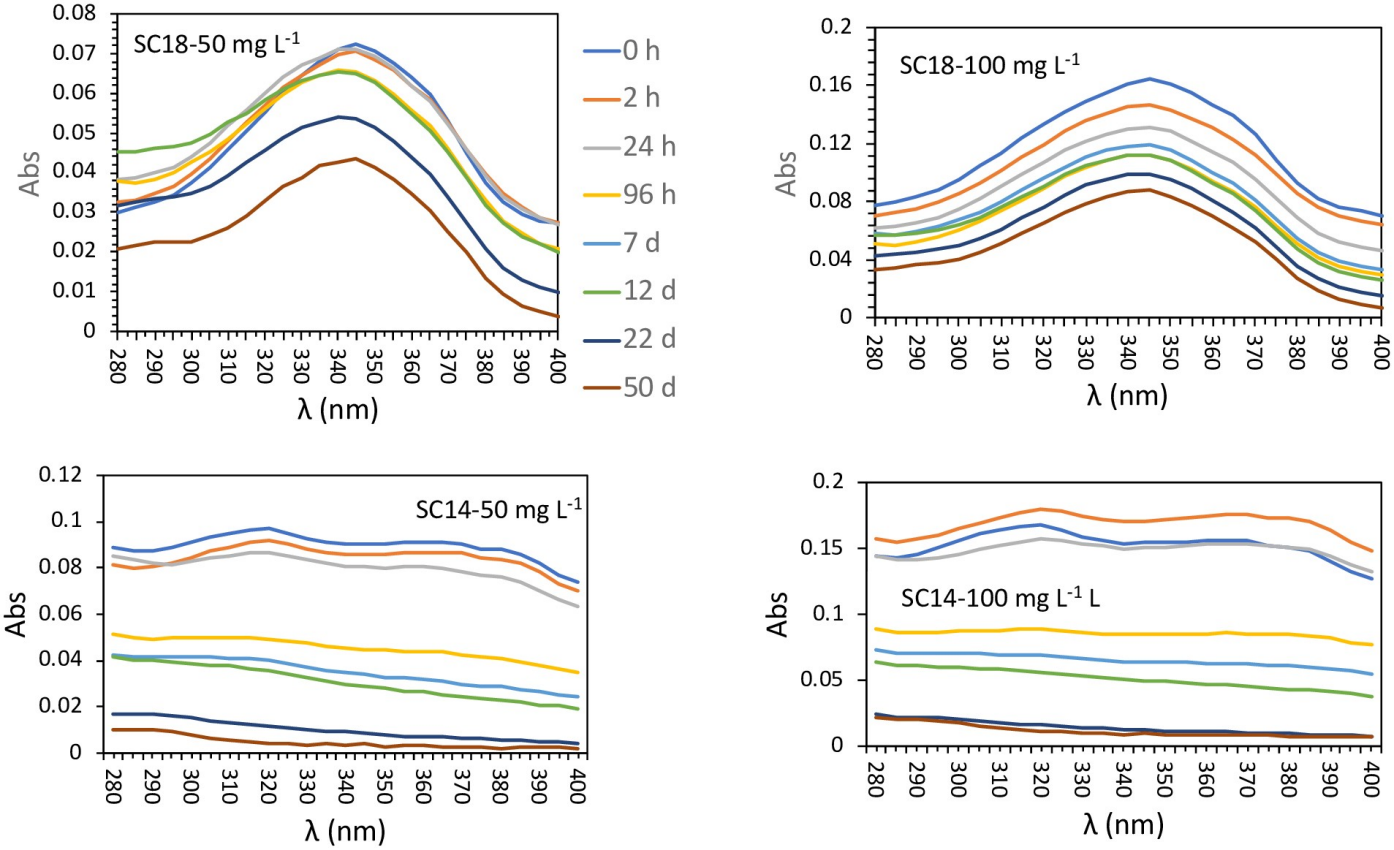
Absorption spectra of sunscreens 14 and 18 at two different concentrations (50 and 100 mg/L), throughout time (from 0 hours to 50 days).

In order to determine for how long sunscreens maintain their absorbance properties in seawater, we measured the spectra of absorbance in the range of UVA and UVB wavelengths (280–400 nm) of 2 different sunscreens (SC14 and SC18) at the concentrations of 50 and 100 mg L^-1^ (Fig 4) throughout time (up to 50 days). Although variable among the two sunscreens, samples (stored at room temperature) kept their spectroscopic properties in the range of 300–305 nm for up to 24 hours (RSD for SC14 of 4.6% and 5.8% for 50 and 100 mg L^-1^, respectively and 8.6% and 11.8% for 50 and 100 mg L^-1^, respectively for SC18). Absorbances decreased throughout time dropping up to 92% (for 50 mg L^-1^) and 90% (for 100 mg L^-1^) in SC14 and 40% (for 50 mg L^-1^) and 57% (for 100 mg L^-1^) in SC18 after 50 days. This information is useful methodologically but might also be relevant from an environmental point of view since it suggests that sunscreens could remain chemically active in the sea for relatively long periods of time. Information on the residence time of sunscreen in the sea is crucial to evaluate the risk of bioaccumulation and toxicity for marine organisms.

### 3.4 Sunscreen quantification in real samples

**Fig 5:**
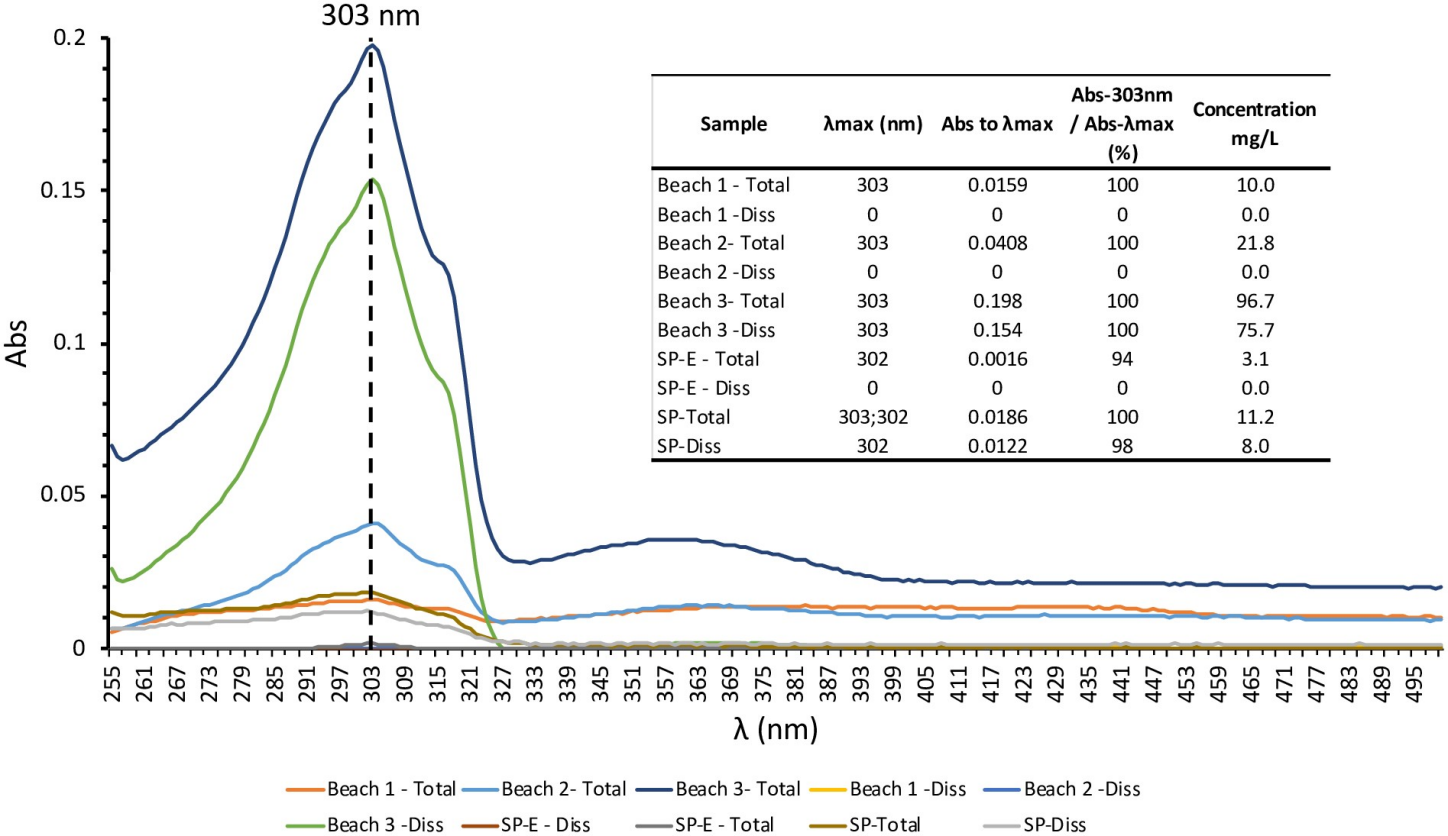
Absorption spectra of samples collected June 28^th^, 2020, from an Atlantic beach (“beach” samples) and in a swimming pool with bathers (SP) and empty of bathers (SP-E) in both, filtered (diss) and unfiltered (total) fractions. Dashed line indicates the 303 nm wavelength. Embedded Table shows, for each sample, the wavelength of maximum absorbance (λmax), the percentage of absorbance measured at 303 nm regarding maximum absorbance and the calculated concentrations using the external calibration curve.

Three dissolved (<0.45 μm) and total (unfiltered) surface (5 cm) seawater samples were analyzed using the method proposed at an Atlantic beach under two different conditions: without and with beachgoers (May 28^th^ and June 28^th^, respectively). No absorbance peak for any seawater fraction was detected in the absorption spectra of samples collected in May when the beach, due to the COVID-19 pandemic situation in Spain, was closed to the public. However, in June, with the beach open and in the presence of beachgoers, maximum peaks of absorbance were found at 303 nm in both seawater fractions (Fig 5). The quantification of sunscreen using a calibration curve prepared with the sunscreen standard stock and measured at 303nm wavelength showed a higher average concentration in the total fraction (42.8 ± 47.0 mg L^-1^) than in the dissolved fraction (25.2 ± 43.7 mg L^-1^) (Fig 5). This finding agrees with previous studies where UV filter concentrations in the total fraction of seawater are higher than the dissolved fraction, probably due to the accumulation of these chemicals on suspended particles [22]. A simple and conservative calculation seems to validate the magnitude of the sunscreen concentrations measured. We considered a volume of seawater affected by sunscreens of 15 m^3^ resulting from a bathing area of 3000 m^2^ (Fig 1) and the 5 cm depth sampled that is the layer where sunscreens are mainly concentrated [5]. With approximately 100 beachgoers in the water during sampling and assuming that a typical application of sunscreen by an adult is approximately 18 g/application and that 25% of sunscreen applied could be washed off the skin into the water [18]; we estimate 450 g of sunscreens released, which results in an estimated concentration of 30 mg L^-1^ which is of the same order as the previously measured sunscreens.

Validation of our method with a gold-standard methods (e.g. mass spectrometry) is not possible because they are selectivity for a single chemical. In order to preclude the possibility that other compounds present in the complex seawater matrix (such as dissolved and particulate organic matter, salt components, lithogenic material, etc.) were absorbing at the target wavelength of 303 nm, we analyzed the absorption spectra of 2 samples (with and without bathers) in both fractions (dissolved and total) from a swimming pool. Even with a completely different matrix the absorption spectra were similar to those of seawater with the maximum peaks of absorbance also found at 303 nm (and 302 nm) in both water fractions (Fig 5), confirming that the absorbance measured at 303 nm is mainly due to sunscreens. Although this was not the main objective of this study, the method was applied to quantify the concentration of sunscreens in the swimming pool. In this case, concentrations of sunscreens with bathers increased almost 4 times (from 3.1 to 11.2 mg L^-1^) in unfiltered samples and from undetectable to 8 mg L^-1^ in the dissolved fraction.

## 4. Conclusions

The spectrophotometric method proposed requires no sample pretreatment, allows the determination at 303 nm of sunscreens in the range of 2.5–1500 mg L^-1^, it offers a LOD of 2.5 mg L^-1^ and a range of precision of 0.2–1.4%. The method was applied to quantify sunscreen concentrations at an Atlantic Beach with values ranging from 10 to 96.7 mg L^-1^ in the unfiltered fraction and from the undetectable value to 75.7 mg L^-1^ in the dissolved fraction. It has been demonstrated that sunscreens, emerging pollutants used by modern society, affect coastal marine ecosystems [1] in several ways. Nowadays these cosmetics are some of the main environmental concerns because of their toxic effects on fragile and protected marine ecosystems such are coral reefs. It is therefore a priority to be able to detect and monitor the environmental levels of these cosmetics in order to help to establish environmental management actions. The proposed method can be used as an effective tool for this purpose, since it is selective, simple and cheap (based in spectrophotometric absorbance), fast and clean (it can be applied to in-situ measurement, does not require reagents and does not generate waste) and sensitive (if needed, sensitivity could be much improved using a larger path length or a fiber optic spectrometer with flow cells).
